# On Three-Dimensional Flow and Heat Transfer over a Non-Linearly Stretching Sheet: Analytical and Numerical Solutions

**DOI:** 10.1371/journal.pone.0107287

**Published:** 2014-09-08

**Authors:** Junaid Ahmad Khan, Meraj Mustafa, Tasawar Hayat, Ahmed Alsaedi

**Affiliations:** 1 Research Centre for Modeling and Simulation (RCMS), National University of Sciences and Technology (NUST), Islamabad, Pakistan; 2 School of Natural Sciences (SNS), National University of Sciences and Technology (NUST), Islamabad, Pakistan; 3 Department of Mathematics, Quaid-I-Azam University, Islamabad, Pakistan; 4 Nonlinear Analysis and Applied Mathematics (NAAM) Research Group, King Abdulaziz University, Jeddah, Saudi Arabia; China University of Mining and Technology, China

## Abstract

This article studies the viscous flow and heat transfer over a plane horizontal surface stretched non-linearly in two lateral directions. Appropriate wall conditions characterizing the non-linear variation in the velocity and temperature of the sheet are employed for the first time. A new set of similarity variables is introduced to reduce the boundary layer equations into self-similar forms. The velocity and temperature distributions are determined by two methods, namely (i) optimal homotopy analysis method (OHAM) and (ii) fourth-fifth-order Runge-Kutta integration based shooting technique. The analytic and numerical solutions are compared and these are found in excellent agreement. Influences of embedded parameters on momentum and thermal boundary layers are sketched and discussed.

## Introduction

The fundamental problem of two-dimensional flow due to stretching plane surface, initially discussed by Crane [1], is involved in various industrial processes such as metal and polymer extrusion, drawing of plastic films, paper production etc. Owing to such applications, the researchers have discussed this problem under various aspects including suction or injection, variable surface temperature, convective boundary condition, mass transfer, mixed convection etc. The three-dimensional flow due to plane bi-directional linearly stretching sheet was first discussed by Wang [2]. He found an exact similarity solution of the classical Navier-Stokes equations. Later, Lakshmisha et al. [3] numerically examined the unsteady three-dimensional flow with heat and mass transfer over an unsteady stretching sheet. In contrast to this problem, Takhar et al. [4] investigated the three-dimensional flow of an electrically conducting fluid due to an impulsive motion of the stretching sheet. Ariel [4] derived approximate analytic and numeric solutions for steady three-dimensional flow over a stretching sheet. Xu et al. [5] provided uniformly valid series solutions for three-dimensional unsteady flow caused by the impulsively stretching sheet. Liu and Andersson [6] considered the heat transfer in three-dimensional flow due to non-isothermal stretching sheet. The unsteady three-dimensional flow of elastico-viscous fluid and mass transfer due to unsteady stretching sheet with constant wall concentration was studied by Hayat et al. [7]. In another paper, Hayat et al. [8] described the three-dimensional flow of Jeffrey fluid due to stretching sheet. Liu et al. [9] firstly discussed the three-dimensional flow due to exponentially stretching sheet numerically. Steady flow of nanofluid past a linearly bi-directional stretching sheet through Buongiorno's model was examined by Junaid et al. [10]. Sheikholeslami and Ganji [11] discussed the flow and heat transfer of nanofluid between parallel sheets in the presence of Brownian motion and thermophoresis effects.

The literature cited above deals only with the case of either linearly or exponentially driven velocity of the sheet. Vajravelu [12] numerically discussed the viscous flow due to stretching sheet when the velocity of the sheet was assumed to obey the power-law distribution, i.e.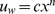. He computed numerical solutions for various values of power-law index 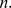 Cortell [13] extended this problem by considering viscous dissipation effects and variable surface temperature. The steady boundary layer flow of micropolar fluid over non-linearly stretching sheet was discussed by Bhargava et al. [14]. Radiation and viscous dissipation effects on the boundary layer flow above a non-linearly stretching sheet were explored by Cortell [15]. Homotopy analytic solutions for mixed convection flow of micropolar fluid past a non-linearly stretching vertical sheet were obtained by Hayat et al. [16]. Kechil and Hashim [17] derived analytic solutions for MHD flow over a non-linearly stretching sheet by Adomian decomposition method. Hayat et al. [18] used modified decomposition method for the series solutions of MHD flow of an electrically conducting fluid over a non-linearly stretching surface. The impact of chemical reaction on the flow over a non-linearly stretching sheet embedded in a porous medium was investigated by Ziabakhsh et al. [19]. Rana and Bhargava [20] computed numerical solutions for two-dimensional flow of nanofluid due to non-linearly stretching sheet by finite element method. Shahzad et al. [21] obtained closed form exact solutions for axisymmetric flow and heat transfer when the velocity of the stretching sheet was proportional to 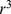. Partial slip effects on the boundary layer flow past a non-linearly permeable stretching surface have been addressed by Mukhopadhyay [22]. In another paper, Mukhopadhyay [23] analyzed the flow and heat transfer of Casson fluid due to non-linearly stretching sheet. Rashidi et al. [24] derived homotopy based analytic solutions for flow over a non-isothermal stretching plate with transpiration.

To our knowledge, the three-dimensional flow due to non-linearly stretching sheet has not been yet reported. It is obvious that three-dimensional flows can be appropriate in giving more clear physical insights of the real world problem when compared with the two-dimensional flows. The present work is therefore undertaken to fill such a void. The study also assumes that the temperature across the sheet is non-linearly distributed. Introducing a new set of similarity variables the boundary layer equations are first reduced into self-similar forms and then solved both analytically and numerically. It is pertinent to mention that computation of either approximate analytic or numerical solutions of the boundary layer equations governing the flow and heat transfer is often challenging (see [25]–[33] for details). Attention is focused on the physical interpretation of parameters including the power-law index 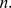


## Mathematical Modeling

**Figure 1:**
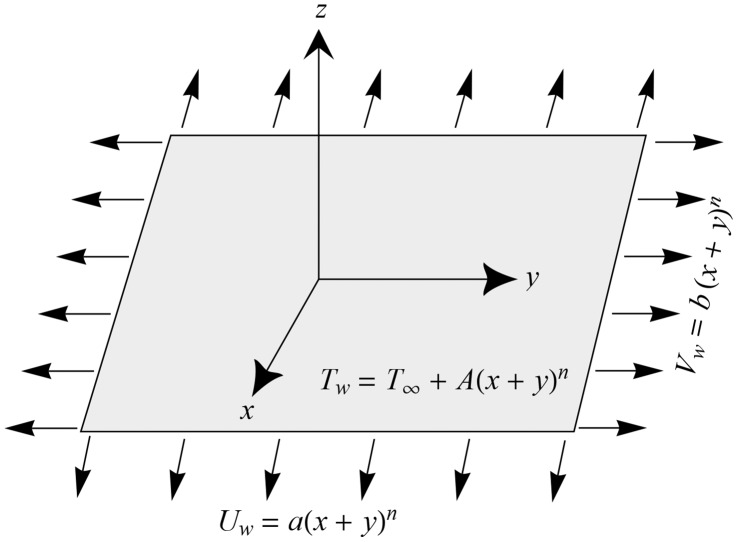
Physical configuration and coordinate system.

Let us consider the three-dimensionalincompressible flow over a plane elastic sheet located at 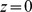 as shown in the Fig. 1. The flow is induced due to stretching of the sheet in two lateral directions. Let 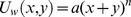 and 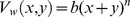 be the velocities of the sheet along the 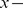 and 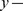 directions respectively with 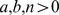 are constants (see Table 1). 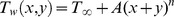 is the variable surface temperature where 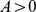 is constant and 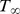 is the ambient fluid temperature. Under the usual boundary layer assumptions, the equations governing the three-dimensional flow and heat transfer in the absence of viscous dissipation and internal heat generation/absorption can be expressed as (see Liu et al. [9])
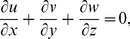
(1)


**Table 1:** List of symbols.

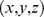 Cartesian coordinate system	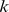 thermal conductivity
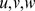 velocity components along the 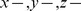 directions	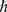 non-zero auxiliary parameter
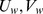 velocity of the stretching sheet along 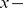 and 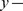 direction	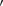 1^st^ order derivative with respect to 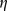
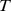 fluid temperature	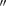 2^nd^ order derivative with respect to 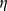
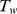 wall temperature	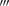 3^rd^ order derivative with respect to 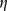
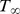 ambient fluid temperature	***Greek symbols***
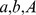 positive constants	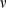 kinematic viscosity
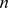 Power-law index	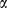 thermal diffusivity
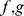 dimensionless stream function	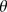 dimensionless temperature
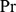 Prandtl number	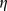 similarity variable
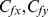 skin friction coefficient along 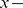 and 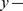 direction	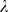 ratio of the stretching rates
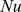 local Nusselt number	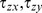 wall shear stress along 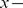 and 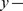 direction
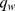 wall heat flux	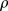 density of the fluid
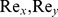 local Reynolds number along 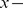 and 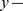 direction	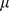 dynamic viscosity



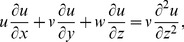
(2)




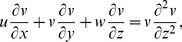
(3)




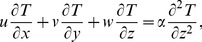
(4)where 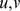 and 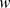 are the velocity components along the 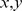 and 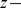 directions respectively, 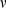 is the kinematic viscosity, 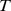 is the fluid temperature and 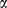 is the thermal diffusivity (see Table 1). The boundary conditions are imposed as under:



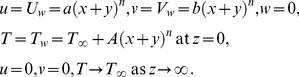
(5)


We introduce the new similarity transformations as follows:
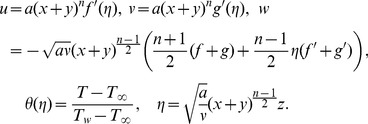
(6)


We have modified the similarity transformations used by Liu et al. [9] for the current problem. Using (6), Eq.(1) is identically satisfied and Eqs. (2)–(5) become
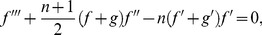
(7)




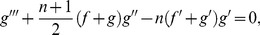
(8)




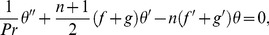
(9)




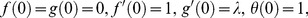
(10)




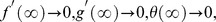
where 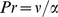 is the Prandtl number and 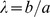 is the ratio of stretching rate along the 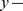 direction to the stretching rate along the 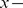 direction (see Table 1). The above equations reduce to the case of two-dimensional flow when 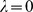. Moreover, when 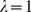, the equations governing the axisymmetric flow due to non-linearly stretching sheet are recovered. When 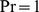 the solution of 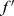 is also a solution of 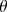. The quantities of practical interest are the skin friction coefficients and the local Nusselt number which are defined as below:
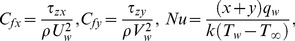
(11)where 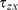 and 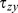 are the wall shear stresses and 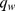 is the wall heat flux. These are given by



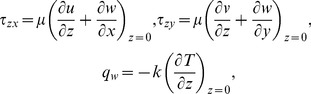
(12)


using Eqs. (6) and (12) in Eq. (11), one obtains
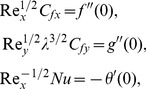
(13)where 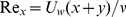 and 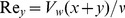 are the local Reynolds numbers along the 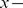 and 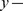 directions respectively (see Table 1). The vertical component of velocity at the far field boundary can be expressed as


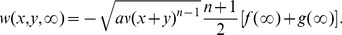
(14)

## Optimal homotopy analytic solutions

The non-linear differential equations (7)–(9) with the boundary conditions (10) have been solved by optimal homotopy analysis method (OHAM) [34], [35]. For this purpose, we first select the initial guesses 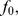 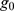 and 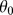 of 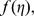 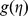 and 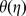 as under:
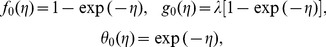
(15)


and the auxiliary linear operators are selected as below
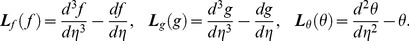
(16)


If 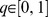 is the embedding parameter and 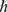 the non-zero auxiliary parameter, then the generalized homotopic equations corresponding to (7)–(10) can be written as follows
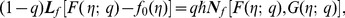
(17)




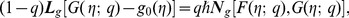
(18)




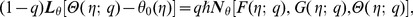
(19)




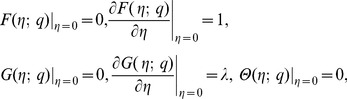
(20)




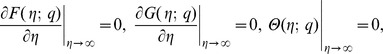
where the non-linear operators 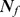, 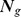 and 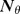 are



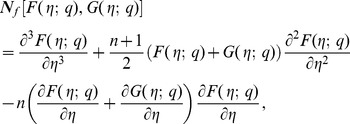
(21)

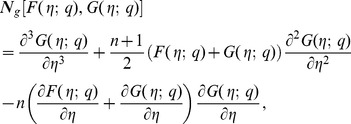
(22)




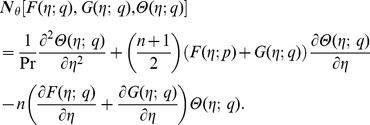
(23)


By Taylor's series expansion one obtains
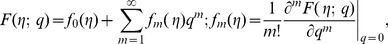
(24)




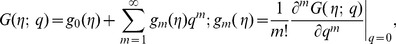
(25)




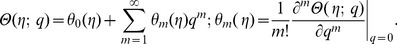
(26)


Substituting 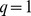 in the above equations yields the final result. The functions 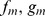 and 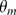 can be determined from the deformation of Eqs. (7)–(10). Explicitly the *m*th-order deformation equations corresponding to Eqs. (7)–(10) are as below
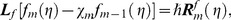
(27)




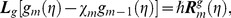
(28)




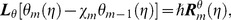
(29)




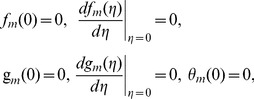
(30)




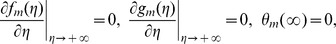





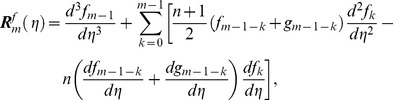
(31)




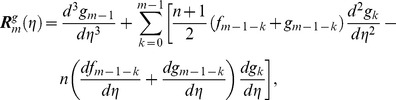
(32)




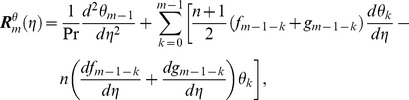
(33)




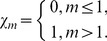
(34)


In order to determine the optimal values of 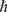 we define the squared residuals of the governing Eqs. (7)–(10), 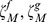 and 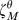 as
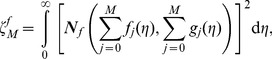
(35)




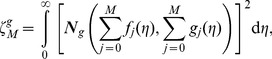
(36)




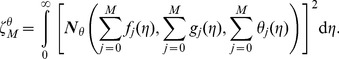
(37)


Such kind of error has been considered in other works [36]–[41]. The smaller 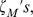 the more accurate the mth order approximation of the solution. The optimal values of 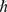 can be obtained by minimizing the 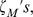 through the command *Minimize* of the software MATHEMATICA (see Liao [36] for details). Alternatively MATHEMATICA package bvph 2.0 can also be used to calculate such values (see [41] for details).

## Numerical method

Eqs. (7)–(9) subject to the boundary conditions (10) have been solved numerically by shooting method with fifth order Runge-Kutta integration procedure. First, we reduce the original ODEs into a system of 1^st^ order ODEs by substituting 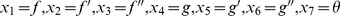 and 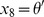 which gives
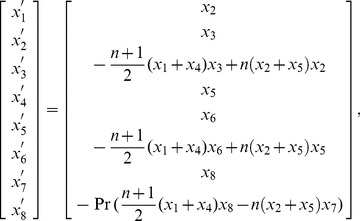
(38)


and the corresponding initial conditions are
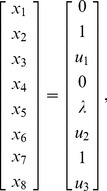
(39)


Suitable values of the unknown initial conditions 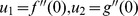 and 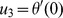 are guessed and then integration is carried out. The values of 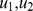 and 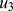 are then iteratively computed through Newton's method such that the solutions satisfy the boundary conditions at infinity (given in Eq. (10)) with error less than 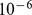.

## Results and Discussion

**Figure 2:**
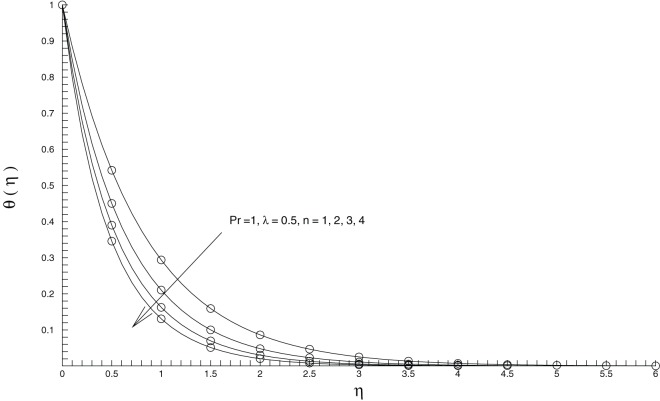
Comparison of analytical and numerical solutions for the temperature distribution. Lines: 15^th^-order OHAM solutions, Circles: Numerical solution.

This section contains the physical interpretations of the behavior of the interesting parameters entering into the problem. We compare the 15^th^-order OHAM solutions for temperature 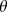 with the numerical ones for different values of 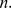 Fig. 2 shows that data retrieved from both solution methods are identical, demonstrating the validation of our findings.

**Figure 3:**
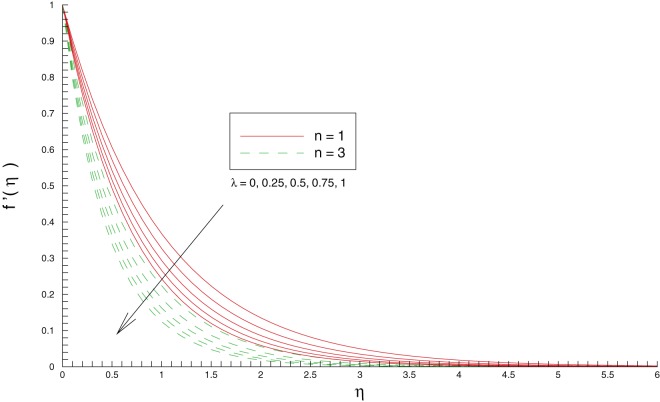
Influence of stretching rates ratio 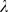 on the 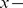 component of velocity 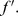

**Figure 4:**
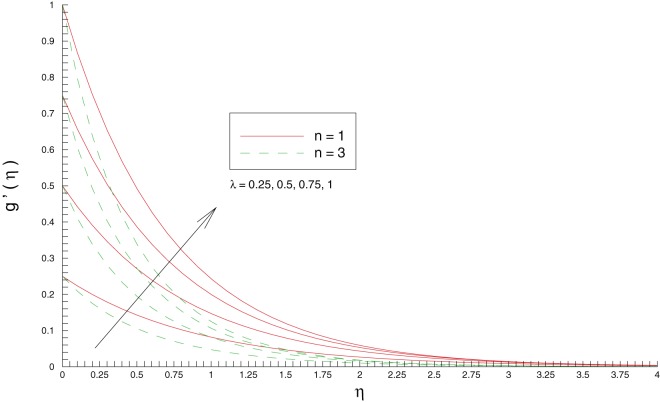
Influence of stretching rates ratio 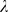 on the 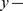 component of velocity 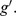

**Figure 5:**
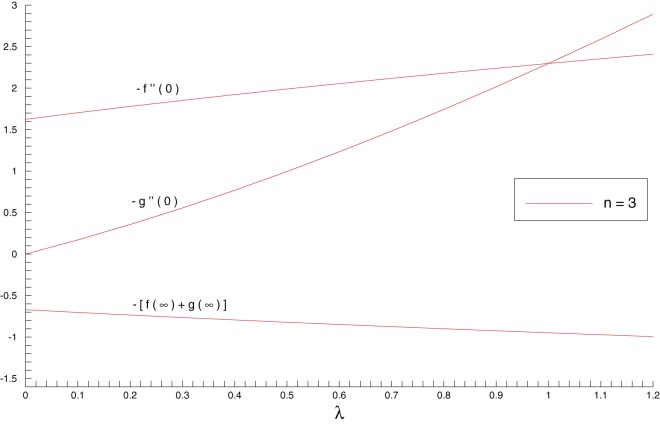
Influence of stretching rates ratio 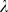 on the skin friction coefficients 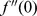 and 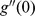 and entrainment velocity 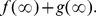

Figs. 3 and 4 show the variations in horizontal and vertical components of velocity with an increase in stretching rates ratio 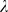. It is clear that increase in 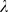 corresponds to an increase in the stretching rate along the 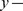
direction. Due to this reason the vertical component of velocity increases with an enhancement in 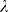 while the velocity in the 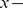
direction decreases correspondingly. The wall velocity gradients 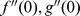 and entrainment velocity 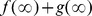 as functions of stretching rates ratio 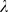 have been plotted in Fig. 5. Due to the bi-directional stretching sheet, there will be downward flow in the vertical direction. The vertical component at far field boundary is therefore expected to be negative in this situation. We notice that shear stresses at the wall increase when 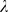 is increased. Further, the larger values of 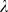 enhances the velocity of the cold fluid at the ambient. As a consequence, the entrainment velocity is an increasing function of 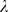.

**Figure 6:**
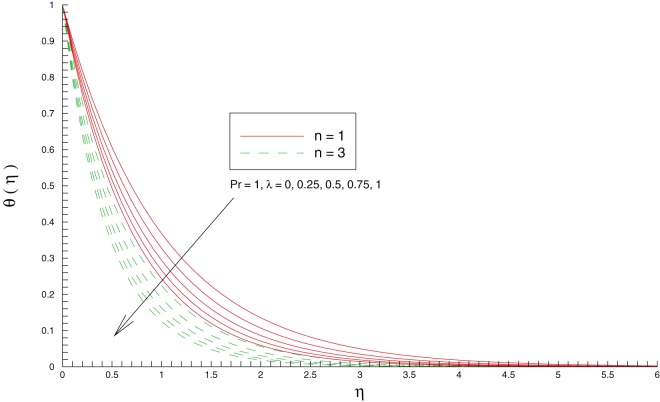
Influence of stretching rates ratio 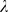 on the temperature 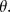

**Figure 7:**
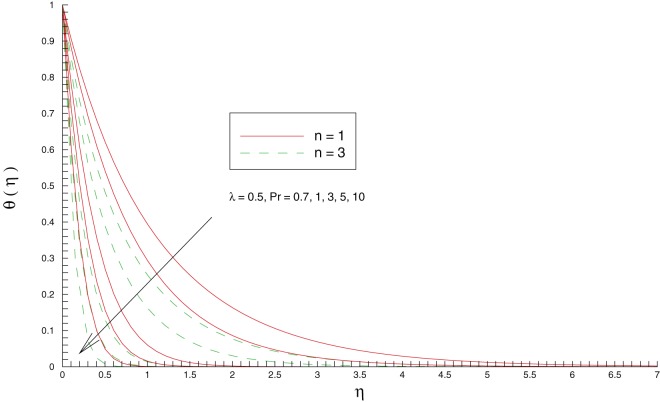
Influence of Prandtl number 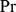 on the temperature 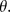

Fig. 6 indicates that temperature 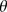 decreases with an increase in stretching rates ratio 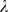 for unity Prandtl number. Physically, an increase in 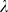 enhances the intensity of colder fluid at the ambient (as noticed in Fig. 6) towards the hot sheet which eventually corresponds to decrease the local fluid temperature. Fig. 7 perceives the behavior of Prandtl number 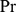 on the temperature. A bigger Prandtl number fluid has less thermal diffusivity and hence it allows less thermal effect to penetrate deeper into the fluid. As a result, temperature decreases and the thermal boundary layer becomes thinner when 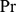 is increased. This decrease in thickness of the thermal boundary layer is compensated with a larger wall slope of temperature function.

**Figure 8:**
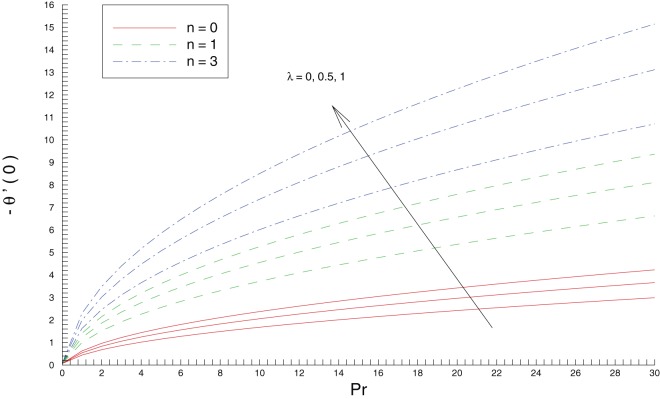
Influence of Prandtl number 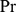 and stretching rates ratio 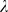 on the wall temperature gradient 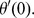

Fig. 8 plots the wall temperature gradient against 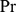 with the variation in stretching rates ratio 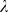. The wall heat transfer rate approaches the zero value for vanishing Prandtl number 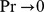, a fact that is clear from the energy equation (9). Moreover, this Fig. compliments the results of Fig. 4. In bigger Prandtl number fluids the convection is effective in transferring energy from the stretching sheet compared to pure conduction. Due to this reason the wall heat transfer rate is an increasing function of 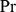. The reduction in thermal boundary layer thickness with an increase in 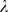 meets with the bigger magnitude of local Nusselt number. In other words the enhanced intensity of cold fluid at the ambient towards the hot fluid closer to the sheet results in larger heat transfer rate at the sheet.

Tables 2 and 3 provide the numerical values of skin friction coefficients and local Nusselt number for different values of parameters by employing shooting method. The results are compared with the MATLAB built in function bvp5c and found in excellent agreement. We notice that wall shear stresses increase with an increase in 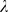 more rapidly at 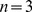 when compared with 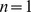. The thinner thermal boundary layer accounted for larger 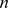 accompanies with larger temperature gradient along the sheet. The magnitude of increase in wall temperature gradient 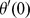 with an increase in 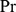 increases when 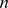 is increased.

**Table 2:** Numerical values of 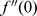 and 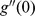 for different values of 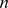 and 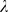.

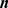	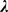	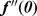		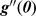	
		**shooting**	**bvp5c**	**shooting**	**bvp5c**
1	0	−1	−1	0	0
	0.5	−1.224745	−1.224742	−0.612372	−0.612371
	1	−1.414214	−1.414214	−1.414214	−1.414214
3	0	−1.624356	−1.624356	0	0
	0.5	−1.989422	−1.989422	−0.994711	−0.994711
	1	−2.297186	−2.297182	−2.297186	−2.297182

**Table 3:** Numerical values of local Nusselt number 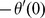 for various values of 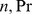 and 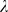.

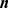	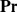	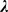	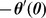	
			**shooting**	**bvp5c**
1	0.7	0	0.793668	0.793668
		0.5	0.972033	0.972029
		1	1.122406	1.122321
	1	0	1.000000	0.999990
		0.5	1.224745	1.224742
		1	1.414214	1.414214
	7	0	3.072250	3.072251
		0.5	3.762723	3.762724
		1	4.344818	4.344779
3	0.7	0	1.292193	1.292194
		0.5	1.582607	1.582607
		1	1.827437	1.827427
	1	0	1.624356	1.624356
		0.5	1.989422	1.989422
		1	2.297186	2.297182
	7	0	4.968777	4.968777
		0.5	6.085484	6.085485
		1	7.026912	7.026913

## Conclusions

For the first time, the flow and heat transfer over a plane surface stretched non-linearly in two lateral directions have been investigated. The simulation in this study assumes that the temperature across the sheet is non-linearly distributed. Both analytic and numerical solutions are obtained and found in excellent agreement. Following are the major results of this study.

I. It is seen that shear stress at the wall increase when the stretching rates ratio is increased. The entrainment velocity is negative, representing a downward flow in the vertical direction, which is a consequence of the bi-directional stretching sheet.
II. The increased intensity of the cold fluid at the ambient towards the stretching sheet with an increase in stretching rates ratio 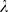 decreases the fluid temperature.
III. The temperature decreases and thermal boundary layer thins when the power-law index 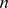 is increased.
IV. The results for the case of two-dimensional and axisymmetric flows can be obtained as special cases of present study when 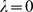 and 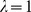 respectively.
